# Bacillus Tuberculosis

**Published:** 1886-08

**Authors:** H. D. Schmidt

**Affiliations:** New Orleans, La.


THE CHICAGO

### MEDICAL JOURNAL AND EXAMINER.

VOL. LIII. AUGUST, 1886. NO. 2.

### ORIGINAL COMMUNICATIONS.

---

VI.

### BACILLUS TUBERCULOSIS. CONCLUSIONS FROM THE RESULTS OF INVESTIGATION. *By* H. D. SCHMIDT, M. D., *of New Orleans, La.*

In reviewing the results of my investigations into the nature of the bacterium tuberculosis and its relation to the human lungs, or other organs, it will be found that, excepting the development of this organism in the nuclei of the tubercle-cells, they correspond, perhaps, in most other points to the statements previously made by other authors. The development of the bacterium tuberculosis in, or, as it almost appears, from the protoplasm of these nuclei is a phenomenon, not only difficult to explain, but which, moreover, may yet bear importantly upon the whole bacterium tuberculosis question. Hence it is worthy to be presented to the reader in its proper light, which, however, involves a review of other obscure points in the history of the bacterium tuberculosis, especially of the manner in which this organism is said to gain access to the human lungs, or other organs.

When Koch, in 1882, announced his discovery of the bacillus tuberculosis, he asserted it to represent the original and special cause of tuberculosis, demonstrating, at the same time, the manner in which it had gained access to the human lungs as to give rise to the tubercle of tuberculosis. His explanation was mainly based upon the supposition, that these special bacteria were constantly floating in the air; and that, accordingly, some, or even one, of them would enter the human lungs during the act of inspiration; and, being carried by the inspiratory current of air to the terminations of the bronchial system, the air-vesicles, settle there upon one or the other epithelial cell, lining these minute cavities; the irritation, set up in the protoplasm of this cell by the presence of the parasite, was to be regarded as the first impulse given to the formation of the tubercle.

It is, particularly, the giant-cells of the tubercle which Koch regards as the special seat of the infectious agent; *for he says, as soon as they make their appearance in the tubercles, bacilli tuberculosis will be regularly met with; if the number of the latter in the tubercle is very limited, they will almost always be found in the giant-cells; and, then, never more than one, or a few individuals in each cell. But when, in correspondence to a more intense course of the process, the bacilli are present in large numbers, then many, fifty or more, will be found enclosed in one giant-cell. The arrangement of these organisms in the giant-cells, Koch states, often takes place in a peculiar manner, namely: If there is only one bacillus enclosed in a cell, it will mostly be found in the centre of the latter, or very little excentrically; but, if the nuclei are lying closely together near, or at one end or pole of the cell, then the bacillus will be found at

* "Die Aetiologie der Tuberculose," von Dr. R. Koch, reviewed by Litten. "Deutsche Medizinal Zeitung," March 24, 1884.

In the month of March, 1885, Dr. James Nevins Hyde, Professor of Diseases of the Skin in Rush Medical College, Chicago, published a paper in the *Chicago Medical Journal and Examiner* entitled "On the Affections of the Skin, Induced by Temperature Variations in Cold Weather." This was followed, in February, 1886, by a second paper, more fully considering the same subject, including the treatment of the so-called "Prairie Itch," "Ohio Scratches," etc.

As both of these papers were soon out of print, the publishers have reproduced the two in a single monograph, with index, which the author has carefully revised in every part, and to which he has made a large number of additions, relating more particularly to the treatment of the class of disorders under discussion.

This will be mailed to any address by the publishers, THE CLARK & LONGLEY CO. 308 Dearborn St., on receipt of the price, 25 cents.

the other end, that is, in that part of the cell which is free from nuclei. This arrangement of the nuclei opposite to the bacillus, Koch regards as a manifestation of an "antagonism," existing between the nuclei of the giant-cell and the parasite therein enclosed, causing the former to get as far as possible away from the latter; even if the bacilli in the giant-cell increase in number, this "oppositional" grouping of the nuclei may still take place, though then, an entirely different arrangement of the bacilli is generally observed. To Koch it almost appears that with the numerical increase of the bacilli, their attitude (*Haltung*) or bearing toward the nuclei becomes more active; for, he says, they then advance more and more toward the periphery of the cells; and, inserting themselves between the nuclei, finally even break through the walls of these bodies. Besides, the destruction of the giant-cell appears to follow regularly such an increase of the bacilli; for very often, in the vicinity of giant-cells containing bacilli arranged in a radiating manner, and in a direction toward the interior of the tuberculous centre, groups of bacilli are met with, which, though not any more enclosed in brown-colored nuclei, still show the same radiating arrangement. As many transitive forms are besides met with, it cannot be doubted that such radiating groups of bacilli indicate the places of former giant-cells, of which the nuclei have disappeared, and of the contents of which only the bacilli have been left.

The above mentioned statements of Koch show that he attaches much importance to the relationship existing between the bacteria tuberculosis and the giant cells. As far as I am able to interpret these statements, he regards the appearance of these giant-cells and that of the tubercle-bacteria as occurring almost simultaneously, though it is not stated whether the bacterium gave rise to the giant-cell, or whether the latter produced the former. If the number of bacteria is very limited, Koch finds them always confined to the giant-cells, but never more than one, or a few individuals in each cell. From this I cannot but infer, that he regards these individuals as the original bacteria, carried by the inspiratory current of air to the interior of the air-vesicles of the lung, where, by irritating the protoplasm of one or the other lining epithelial cells, they give rise to the formation of a giant-cell. Although the exact nature of these giant-cells is as yet not positively known, we shall in this instance, nevertheless, suppose that they represent irritated epithelial cells, the nuclei of which have multiplied by division, while the protoplasm of the cells has simply increased in bulk without division. The inference to be drawn from Koch's observations on the giant-cells would be as follows: The bacterium, after having caused by its presence the multiplication of the original nucleus and the increase of the protoplasm of the cell, quietly remains in one corner of the latter, while the nuclei, being, as it appears, afraid of the parasite, get away as far as possible from their antagonist by withdrawing to the other corner. But, now, the bacterium also commences to increase in number by reproducing its own kind, and, as soon as its descendants are sufficiently numerous, their attitude toward the nuclei becomes more active; for, advancing like so many soldiers, toward the periphery of the cell, they commence the attack upon the nuclei by inserting themselves between these bodies, and finally conquer by breaking through their walls and by settling in their interior. Thus the "antagonism," which Koch supposes to exist between the bacteria and the nuclei of the giant-cell, leads to the destruction of the latter, and to the remaining of the former upon the field of battle.

As far as my own observations extend I may state that I

have never observed the above described phenomenon concerning the bacteria tuberculosis and the nuclei of the giant-cells, which, if true, would almost indicate that these bodies were endowed with a certain kind of instinct, manifesting itself in the " antagonism " to which Koch directs attention. On the contrary, I have met with many giant-cells containing no bacteria tuberculosis, whilst in others I found a smaller or greater number of these organisms enclosed, both in the nuclei and in the protoplasm of the cells. In the same way giant-cells are met with containing a considerable number of nuclei, arranged in line along a part of the periphery of the cell, whilst in others again they are, in smaller or larger numbers, irregularly distributed throughout the protoplasm of the cell. On the whole, I can find no reason for supposing a closer relationship to exist between the giant-cells and the bacteria tuberculosis than between the latter and the other cells of the tubercle. In those instances in which I met with bacteria tuberculosis in the giant-cells I found them either still enclosed in the nuclei or liberated from the latter by the ultimate disintegration of their protoplasm, lying free in the degenerated protoplasm of the cell. But let us now endeavor to find some plausible explanation for the presence of the bacteria tuberculosis in the nuclei of the tubercle cells, in which, as I have shown before, they make their first appearance, and to which they may gain access by different means and ways. Before, however, entering upon this discussion I shall take the liberty of quoting from Litten's review, already mentioned, Koch's views on the subject. They are as follows: "The appearance of one or some bacilli in the interior of cells, bearing an epithelioid character, must be regarded as the first stage of the origin of the tubercle. The manner in which the bacilli get into these cells can hardly be explained in any other way but that, not being themselves endowed with spontaneous motion, they are either in the current of the blood or lymph or in the tissues of already existing tuberculous centres, taken up and carried along by tissue-elements which possess this kind of motion, as, for instance, the wandering cells. The peculiar fact that single bacilli, or small groups of them, separated from each other by one and the same, though comparatively far, distance, are frequently met with, can only be explained in this manner. Charged with such parasites, the cell is still capable of traveling considerable distances, but under the deleterious influence of the bacillus it undergoes changes which will soon cause it to stop. Whether, now, one wandering cell is destroyed and the bacilli are taken up by other cells present at the same place, and which subsequently assume an epithelioid character,—or whether, what Koch thinks to be more probable, the wandering cell, transporting the bacillus is transformed into an epithelioid cell and from this into a giant-cell must, for the present, remain undecided. In order to presume that the bacilli are originally carried along by the wandering cells and that in this manner their distribution in the tissues is effected, the following reasons may be adduced. First, the analogy with the septicæmia of the mice, in which also rod-shaped bacilli are incorporated by colorless blood-corpuscles; furthermore, the direct observation, showing that the tubercle bacilli are first taken up and transported by the wandering cells. This is best recognized in those cases in which large numbers of bacilli are introduced into the circulation, as, for instance, by injection into the veins of the ear of the rabbit. If an animal, infected in this manner, is killed at an early time, numerous leucocytes enclosing one or some bacilli are then still found in the blood; whilst even in the tissues of the lung, liver and spleen true round cells now and then appear which, containing a single or divided nucleus, show as yet no epithelial form; that is to say, they resemble in every respect colorless blood-corpuscles, though containing tubercle bacilli. The same phenomena were observed in guinea-pigs, into the abdominal cavity of which large numbers of tubercle bacilli were injected, and which died already during the course of the first week. A third reason for the above mentioned supposition may be found in completely dead tissues, that is, in such places where the influence of living cells upon the bacilli is entirely excluded; for if here a vigorous growth of bacilli once more occurs, they will arrange themselves in entirely typically formed groups resembling the peculiar forms of bacteria-colonies in the pure culture upon the serum of blood. These forms have to be regarded as those assumed by the tubercle bacilli when developing without disturbance, and when their grouping is solely determined by those local changes of position which depend upon their growth. Every other arrangement must be regarded as caused by some other disturbance. If a too rapid increase of the bacilli within the giant-cells takes place, the latter undergo destruction in being to a certain extent burst by the bacilli pressing themselves between the walls of the nuclei. The nuclei disintegrate into minute granules, and the cell is destroyed. The further changes, taking place in the tuberculous tissues after the development of the epithelioid and the giant cells, are all of a retrograde nature. The greater part of them belongs to those processes known as coagulation-necrosis, and lead to the death of the tuberculous tissues, and to the formation of the so-called cheesy masses which are so frequently found in tuberculous centres. Generally the tubercle-bacilli disappear very soon in the cheesy masses, so that they are only met with in the younger centres (Herden), while they are almost always absent in the older ones. In other cases, however, a simple shriveling and transformation of the tuberculous tissue into a firm connective tissue may take place after the disappearance of the bacillus-vegetation."*

Now let us briefly review the above quotation from which we gather that the appearance of one, or some bacteria tuberculosis in the interior of cells bearing an epithelioid character, is regarded by Koch "as the first stage of the origin of the tubercle," and that, furthermore, the manner in which the bacteria get into these cells can hardly be explained in any other way but that they themselves, being incapable of moving, must be taken up—either in the current of blood or lymph, or in the tissues of already existing tuberculous centres —and carried along by other tissue elements which possess spontaneous motion, such as the wandering cells. The conjecture concerning the transportation of the bacteria tuberculosis by means of wandering cells, appears to me very plausible, especially since I have observed the same phenomenon in the blood of lepers, where I found the bacteria lepræ adhering to the remains of colorless blood corpuscles. But with this conjecture the presence of these organisms in the tubercles of tuberculosis, and particularly in the nuclei of the tubercle cells, is as yet not explained, even not in accepting the theory of the tubercle being originally formed from wandering cells, derived from the blood or lymph, for it is obvious that the bacteria, in order to be taken up by these cells, must already be present either in these fluids, or in already existing tubercles. But as to the particular manner in which the bacteria first gain access to these fluids, or to the parenchyma of the lungs, nothing is said in this place; and, as no other satisfactory explanation on this point can be found in Litten's review

* Page 261.

of Koch's paper, I propose to discuss the different ways and manners in which the bacteria might possibly get into the tissues of the human lungs, or other organs.

The bacteria tuberculosis, being incapable of moving spontaneously, must of course be transported from one place to another by some other agent or moving power. If, therefore, they are, as Koch asserts, constantly present in the air we breathe, it is but natural to suppose that one, or some of them might be carried, as once before mentioned, with the inspiratory current of air into the interior of the alveolar cavities of the lungs, and settle there upon some of the lining epithelial cells. Having settled upon one of these cells, however, the bacterium being motionless, is incapable of penetrating through the protoplasm of the cell into the nucleus, except by decomposing and absorbing the protoplasm before it. This phenomenon is observed among some of the more highly organized fungi, as, for instance, with the so-called potato-fungus (*Peronospora infestans*), which, though being, like the bacterium tuberculosis, incapable of moving spontaneously, nevertheless penetrates not only into the cells of the potato, but, moreover, even into the starch-granules contained therein. The penetration in this case, however, is solely effected by the inherent growth of the fungus, that is, by the lengthening of its filaments, which, in channeling their way into the interior of the cell, absorb a part of its wall. Thus, the further the fungus penetrates, the more its filaments gain in length.

Although it is not very probable, I will, for the sake of argument, suppose that the bacterium tuberculosis, after having settled upon the epithelial cell penetrates in the above-mentioned manner into the interior of the nucleus, whilst it, at the same time, increases in numbers; in this way it might even penetrate from cell to cell. But instead of proceeding with our supposition, let us now direct our attention to the tubercle, which, according to Koch, has to originate in the epithelial cell, irritated and already partly destroyed by the bacteria. The tubercle, as we know, can only be formed by the multiplication of one or more cells, brought about by the successive division of the nucleus and the protoplasm of the original cell. The question, therefore, arises: Is a cell, which is already partly destroyed by bacteria, still capable of multiplying; or does it still possess sufficient vitality to give rise to the hundreds or thousands of cells of which a tubercle is formed? For my own part, I cannot but answer the question in the negative. But in admitting even the possibility of the tubercle arising from an epithelial cell, already crippled by bacteria, there are besides other phenomena remaining to be satisfactorily explained. One of these is the simultaneous origin of great numbers of tubercles throughout one, or even both lungs, as is frequently witnessed in acute miliary tuberculosis. In such a case, it is quite difficult to suppose that so many bacteria, as there are tubercles, had simultaneously entered the lungs, and that—while passing through the numerous bronchi and bronchioles—they should distribute themselves so nicely as to settle only in the interior of certain alveoli throughout the whole organ, separated from one another by certain, though frequently quite small, distances, as found between the respective tubercles supposed to be called forth by these bacteria. The appearance of tubercles, separated from one another by greater or lesser distances, has been explained by supposing that the bacteria are transported from one part oi the lung to the other by means of the expectoration; that is to say, by the matter derived from a small cavity, and charged with bacteria, passing outward through one of the smaller bronchial tubes into the larger parent tube, and then returning through another branch of the latter to the terminal lobules of another part of the organ. In answer to this supposition we may say, that it is possible but not very probable, as the normal direction of the secretion is from the smaller bronchi toward the larger ones, the same as that of the movements of the cilia of the epithelial cells lining the bronchi, and assisting in the forward movement of the secretion. But the main objection to this supposition will be found in the fact that not unfrequently cases of miliary tuberculosis are met with in which no cavity whatever can be discovered, though innumerable tubercles are disseminated throughout the whole organ.

There is another mode of explaining the transportation of bacteria from one tubercle to a distant part of the lung; it is the same as that used to explain the phenomenon of metastasis observed in certain morbid growths, that is, through the lymphatics. The fact that all tissues contain lymphatic channels or vessels, makes it appear very plausible that certain neoplastic cells might, in one way or the other, get into these channels to be carried by the lymph-current to other parts of the same, or even of another organ. Such an occurrence, it is true, may happen, but not as easily as is generally supposed.

The lymphatics commence, as we know, in the so-called lymph-spaces, representing minute, irregularly-shaped channels extending, especially through the connective tissues, where they are identical with the interspaces of the fibrillar bundles of this tissue and lined, though incompletely, by endothelial cells. In these channels the radicals of the lymphatic vessels commence. Each organ generally possesses a deep and a superficial set of lymphatic vessels. The radicals of the deep lymphatics, after having arisen from the lymph-channels, generally follow the minute bloodvessels, and, soon joining one another, form larger lymphatic vessels. By successively continued junctures of the latter, the larger lymphatic vessels are formed, which, also following the bloodvessels, finally leave the organ to join the nearest lymphatic glands. But while the radicals of the deep lymphatics, after arising from the lymph-spaces, soon join one another, to give rise to larger lymphatic vessels, those of the superficial lymphatics first form a network which extends over the surface of the organ, and from which the lymphatic vessels subsequently arise. The lymphatic vessels, leaving the lymphatic glands, form larger vessels which finally join one or the other lymphatic duct. Some communications always exist between the deep and the superficial lymphatic vessels of an organ. All lymphatic vessels are provided with valves, which open in the direction of the lymph current, that is, toward the opening of the lymphatic ducts into the subclavian veins, or, in other words, toward the heart. Even the radicals of the lymphatic vessels possess these valves, the presence of which is indicated by a constriction in the wall of the vessel. Many years ago, I injected the lymphatics of the human liver with Prussian blue and even observed these constrictions on lymphatic vessels, smaller in diameter than the capillaries of the blood. From this we may presume, that the current of lymph can only run in one direction. In the lymphatic network on the surface of an organ the current may run sideways or backward into neighboring radicals, but only to a very small extent, as even here, its general direction is toward the lymphatic vessels arising from this network. The lymph-current, joining the blood-current in the subclavian veins, runs of course in the same direction with this, and is, therefore, controlled and kept in motion by the same power, upon which the circulation of the blood depends, that is, the suction-power of the heart. Hence we may presume that the lymph-current, from its very beginning in the lymph-spaces, cannot deviate from its course, but is bound to take the most direct course toward the heart. For this reason we are justified to presume that, if ever bacteria tuberculosis are taken up by the radicals of the lymphatics of the lung, they will be carried by the lymph-current directly to the heart, and not to a distant terminal lobule of this organ, except through the medium of the circulation of the blood. Having once entered the blood, the bacteria might attach themselves to some of the colorless blood-corpuscles, and pass with these through the walls of the capillaries of one or the other organ, and thus give rise to the formation of new tubercles.

The formation of tubercles in this manner is not impossible, though quite improbable; for if this were the case, bacteria tuberculosis would much more frequently be met with in the blood of tuberculous patients than they have in reality been met with thus far. To the extent of my knowledge, there are only three authenticated cases, reported by Weichselbaum, in which these bacteria were found in the blood after death. In these cases cheesy tubercles were found in the uterine veins and Fallopian tubes, in the pulmonary artery, and around the pudendo-vesical plexus, where they had perforated a vein. In ten other cases Weigert has observed tubercles in the veins, and in two others tubercles in the thoracic duct; while tubercles in the latter locality had been previously observed in one case by Ponfick. In the reports of Weigert's and Ponfick's cases, however, nothing is said of the presence of bacteria tuberculosis in the blood, though, if they had really been present in this fluid, it would be obvious that they had entered it directly with the cheesy matter of the tubercles, and not by way of the lymphatics. Nor can the presence of tubercles in the walls of the veins be regarded as an unusual phenomenon, for they are equally met with in the walls of other vessels, or in the peritoneum and other fibrous structures. I have myself stained the blood of several tuberculous patients by Ehrlich's and Gibbes' methods, but failed to discover any bacteria tuberculosis.

From what I have said above it will be seen, that the theory of the formation of tubercles in the human lungs from one, or several of the lining epithelial cells of the alveoli, previously irritated and crippled by bacteria tuberculosis, is rather resting upon a weak foundation, for if the tubercle was really originating in one or more of these cells, its formation could only be effected by a successive division and multiplication of the latter. In admitting, however, the possibility of the formation of the tubercle in this manner, we must likewise admit that a cell, the nucleus of which is undergoing a process of involution by the presence of bacteria in its protoplasm, is still capable of reproducing its kind. In this case the bacteria would be transmitted from cell to cell by the division of the mother-cells. But, even if this theory regarding the origin of the first tubercle were true, it would still fail to explain the simultaneous arising of hundreds, nay, in many cases, thousands of other tubercles, separated one from another by healthy parenchyma in the same lungs.

In the preceding section of this treatise I have shown that the bacteria tuberculosis first appear in the nuclei of the tubercle cells. Simultaneously, or even previously to the appearance of the bacteria in the nuclei, not only a gradual shriveling in the protoplasm of these bodies, but, in many instances, also a breaking up into several fragments is observed, the whole phenomenon indicating the going on of a process of involution. The same phenomenon is also observed, as I have already stated, in the nuclei of the cells of cancerous, or other neoplastic growth, while they are undergoing a certain kind of degeneration; and, furthermore, in the nuclei of pus-cells and other leucocytes, as, for instance, the exudation cells of croupous pneumonia, or even in the nuclei of the epithelial cells of the bronchioles, met with in phthisical and pneumonic expectorations. In the nuclei of the tubercle cells this process of involution, manifesting itself by the shriveling and breaking up of the protoplasm of these bodies, is accompanied, as I have shown, by the appearance and development of bacteria tuberculosis.

Now the question may be asked: How is it possible for the tubercle to develop, if a tubercle-cell is rendered incapable of multiplying itself, as soon as bacteria appear within its nucleus? This apparent contradiction is easily explained in considering that the bacteria tuberculosis, even in incipient tubercles, do not appear in the nuclei of the younger tubercle-cells, but rather in those of the cells first formed Besides, it is not in the nuclei of all the cells of a tubercle that bacteria will be met with; on the contrary, in every tubercle a considerable number of cells is observed, in which the nuclei present their normal form, and where no bacteria can be demonstrated, even after the most careful staining. I have already stated that the nearer the cells of a tubercle are to its cheesy centre, representing the part of the tubercle first formed, the more highly developed are the bacteria, and *vice versa;* though I must say that, now and then, I have observed bacteria in some of the nuclei of the lining cells of alveoli, adjacent to a tubercle, and apparently free from tuberculous growth. These cases, however, are exceptions to the rule. From these observations we may justly presume, that not all cells of the tubercle are in one and the same condition, but that, on the contrary, one part of them may be normal, while the rest is undergoing a process of involution or degeneration; it is in the nuclei of the latter that the bacteria tuberculosis find the proper soil for their development.

In the preceding pages I have discussed some of the ways and manners in which the bacteria tuberculosis might gain access to the nuclei of the tubercle-cells, while showing at the same time the objections which render these suppositions implausible. But having thus far failed to offer an explanation more satisfactory than those already discussed, the reader would now be justified to ask for the *true* manner in which these bacteria originally get into the interior of these nuclei, where, according to my observations, they are developed. Often, during the course of my investigations, I have put this question to myself, but I must confess that hitherto I have completely failed to answer it satisfactorily, because every attempt to solve this problem leads the inquirer unconsciously to one or the other field of speculation. But, being more accustomed to deal with facts than with theories, or hypotheses, I shall further remain content with my ignorance on this part of the subject, until the facts, which I have observed, will be corroborated and properly explained by other investigators. This, however, will not prevent me from simply pointing out the manner in which alone the origin and development of bacteria in the nuclei of neoplastic cells could be *hypothetically* explained. This is as follows:

The observation of the bacteria appearing in the nuclei, before they are seen in the protoplasm of the cells, without being able to explain, on account of their incapacity of moving spontaneously, the exact manner in which they gained access to these bodies, naturally leads to the suggestion that they must have originated there. The observed fact, however, that these organisms are not present in the nuclei of all the cells of a tubercle, or of any other neoplasm, suggests furthermore, that the condition of the protoplasm of the nuclei containing bacteria, must be different from that of those nuclei containing none; that this is really the case is proved by the difference observed in the morphological appearance of these nuclei, as I have sufficiently demonstrated before. Now, there are two hypotheses which might explain the origin of the bacteria in the substance of the nuclei. The one consists in supposing that certain germs, too minute to be detected by the microscope, are constantly present in the nuclei of the cells of the organism, to which they were carried by the nutritive fluid of the latter, the liquor sanguinis, and incorporated with the normal substances of nutrition contained in the fluid. These germs, then, remaining dormant during the normal condition of the cells and their nuclei, might be developed into bacteria as soon as degeneration of the latter takes place.

The second hypothesis is identical with H. CHARLTON BASTIAN'S theory of *heterogenesis*, according to which low organisms, such as bacteria, may be directly formed from the protoplasm of animal cells, the vital activity of which is on the wane, or, in other words, undergoing involution, as, for instance, is witnessed in the tubercle of tuberculosis. When Bastian advanced this theory, a number of years ago, it met, like his theory of "spontaneous generation," with a great opposition, because it did not correspond with the predominating idea, that such organisms must be the offspring of a parent of their own kind. For my own part I cannot blindly discard the theory of heterogenesis, for the reason, that it does not appear to me entirely impossible, that the normal arrangement of the molecules of the protoplasm of the nucleus of an animal cell might under certain conditions of the organism be altered in such a manner as to correspond with that of the molecules of vegetable protoplasm; nor is it proved that such a molecular change in the protoplasm of an animal cell is altogether impossible. On the contrary, it will appear more probable in considering that a difference already exists in the molecular composition of the protoplasm of the nucleus and that of the body of the cell, manifesting itself by the difference in the reactions of certain agents on these protoplasms, as, for instance, acetic acid dissolving the protoplasm of the body of the cell, while it leaves the nucleus unaffected. Furthermore, in staining animal tissues containing bacteria, as sections of tubercle, with aniline colors by Ehrlich's method, this dissimilarity in the molecular composition of the protoplasm of the nuclei and that of the bodies of the cells is rendered very striking by the fact that, while the latter offers no resistance whatever to the decolorizing action of the acids, but readily parts with the color previously absorbed, the former resists to a certain degree, retaining mostly some of the color, though not as much as the bacteria. The resistance of these nuclei to the decolorizing action of the acids even increases, whenever their normal condition is altered. The phenomena just described certainly indicate a similarity of molecular composition existing in the protoplasm of the nuclei of animal cells and in that of the lowest vegetable organisms, such as the bacteria. And in considering this fact a little more closely, the idea,—that under certain conditions of the cell and its nucleus a certain process of involution is set up, by which the constituent particles of the nucleus might, according to Bastian, "individualize themselves" and grow into lower organisms,—will appear less absurd than it has hitherto been regarded. Besides, if it is possible, as we must presume, that at one time or another in the early history of our globe, higher organisms developed from lower ones, while the latter originally arose from a primitive formless protoplasm, then it may not be impossible that the lowest organisms in nature, represented by the bacteria, might spontaneously arise from the still living protoplasm of the nucleus of an animal cell, which under certain unknown conditions had assumed the nature of vegetable protoplasm. Such a process, if even not probable, is at least possible. In the consideration of a question, like the one before us, however, we should always remember that, though modern science has deeply penetrated into the secrets of nature, many natural processes are still left hidden to us. To these belong the mutual relations of the molecules of animal and vegetable tissues, of which our knowledge is rather deficient, for the reason, that molecules cannot be seen, though it is essential that, in order to render the explanation of those phenomena generally manifested by matter more easy, they should hypothetically exist in our mind. The same is the case with Bastian's theory of "heterogenesis" which, at least in some instances, would satisfactorily explain, as I shall demonstrate directly, the action of certain specific and contagious poisons. To demonstrate this, however, a few remarks on the most important property of the bacterium tuberculosis, namely, that of giving rise to the tubercles of tuberculosis, will be necessary.

Although the question whether the bacterium tuberculosis is in reality the direct inciting cause of tuberculosis in man, is not definitely settled, Koch's discovery of tubercles being artificially produced in the lungs, or other organs of certain animals, by the introduction of this organism into the system of the latter, must at present be regarded as an established fact. The conclusion drawn from this fact is, of course, that the property of producing a specific disease like tuberculosis in the organs of these animals is inherent in the bacterium tuberculosis; though the process by which the presence of this organism gives rise to the formation of tubercles in the respective tissues, is still obscure. If the production of tubercles in these animals were confined to the inoculation of those bacteria contained in tuberculous matter, the phenomenon might be easily explained by presuming this matter, and not the bacteria, to represent the specific poison. But this is not alone the case, for tubercles will still be produced in these animals by inoculating them with very remote generations of those bacteria, obtained by successive artificial cultures of those originally contained in tuberculous matter. Now, as it thus far appears to be proved, that no other species of bacteria, nor any other substance is capable of producing tubercles in an animal, when introduced into its organism by inoculation, the only satisfactory explanation of the phenomenon appears to be by presuming that the bacterium tuberculosis must have acquired this special property of producing tubercles from the tuberculous matter itself, from which it drew its nourishment during its development; that is to say, that during the development of the organism, the constituent molecules of its protoplasm arranged themselves in a similar manner as those of the tuberculous matter, on which it lived, and that it thus acquired the properties of the latter, that is of producing tubercles; and furthermore, that these properties are during the artificial cultivation of the bacteria, transmitted from generation to generation.

According to Koch the tubercle bacteria are entirely dependent upon the human and animal organisms for their existence; or in other words, they are incapable of living or reproducing out side of the latter. With reference to this assertion we may ask: where then did they originally come from, if not from the outside world? The most plausible answer for this question may be found in the theory of "heterogenesis," according to which these organisms would originate in the elements of the specifically diseased organ itself. But the bacteria being derived from the latter, it is but natural to presume that they were also endowed with the specific properties of the diseased matter of this organ, manifesting themselves in producing "*alike*," that is, in calling forth the same pathological conditions as were present in the tissues of the respective organ, from which they were derived. In the same way all other specific contagious diseases may be explained by the theory of "heterogenesis."

Although, as may be gathered from the above, I do not deny the contagiousness of tuberculosis in cases where the specific poison is directly transferred from one organism into another, as is done in all the inoculation experiments performed on animals, it nevertheless appears to me very improbable that the numerous cases of tuberculosis in man should have originated in this manner. On the contrary, it is very doubtful that such numbers of tubercle-bacteria, as would be required to produce all these cases, are contained in the atmosphere. Koch, of course, asserts that these bacteria are derived from the expectorations of tuberculous patients, which are left on the floors of rooms or corridors, or on the ground of the streets, and which after getting dry and pulverized by the shoes of people walking over them, are disseminated throughout the air. In examining this proposition a little more closely, it will be found that, notwithstanding the prevalence of tuberculosis, a very small fraction of all the tuberculous expectoration will have a chance to reach the atmosphere in a pulverized state. In commencing our examination among the better situated classes of society, we will find that the tuberculous patients, for the sake of cleanliness, expectorate into spitboxes instead of upon the floor, and that with the cleaning of these articles of household furniture the expectorations will eventually arrive in the sewers of the street, or in some other channel of drainage. If the patient is confined to bed, he will generally be provided with some sort of a vessel, into which he may expectorate; and if he should even make use of a handkerchief, the expectoration will hardly be allowed to dry and to be pulverized, but rather be submitted to the action of the soap-water in the washtub. The same fate the tuberculous expectorations will meet with in hospitals, though in large public institutions of this kind it may happen, now and then, that a tuberculous patient soils the floors of a corridor with his expectoration, which, however, will hardly be left to dry and to be pulverized. Hence it would be only in the lowest strata of society, where cleanliness is unknown, that tuberculous sputa might reach the atmosphere in the form of powder. In all other cases the bacteria tuberculosis, instead of being disseminated throughout the atmosphere, will ultimately arrive in the gutters and sewers of the street, to be carried to the river or ocean, where they will be incapable of living or thriving, because their existence, according to Koch, solely depends upon the human or animal organism. The same will happen to the tuberculous expectorations left on the pavements of the streets, where they will be washed away by the rains of the season. We may, therefore, presume that, as I have remarked before, but a very small fraction of all the bacteria contained in the expectorations of tuberculous patients, will ever come to float in the atmosphere. But even this small fraction, which under favorable circumstances may come to be suspended in the atmosphere, will have but very narrow chances to reach the interior of the human lungs, for the reason that, being, as just mentioned, incapable of living or of increasing their numbers by reproduction outside of the human or animal organism, they will soon be dispersed and swept away to unknown regions by the winds, or removed from the atmosphere by every rain, and like their brethren, finally arrive in the various channels of drainage.

I might still further enlarge upon this subject, but I trust that the foregoing remarks will suffice to show that the numerous cases of tuberculosis cannot be regarded as having been caused by bacteria tuberculosis floating in the atmosphere. In those rare cases in which the disease has in reality been communicated from one individual to another, it was undoubtedly accomplished by the medium of the expectoration itself, which in the form of spray—as may happen during the act of coughing or speaking or even kissing—issues from the mouth of the tuberculous individual to be inhaled by the lungs of another person. Besides the immunity from tuberculosis, generally exhibited by the resident physicians and nurses of hospitals, is another proof to show that this disease is but rarely communicated by the sole medium of the atmosphere; as an instance of this fact I simply point, as has been done by other authors before me, to the well known statistics of the "Hospital for Consumption," at Brompton, in England.

Notwithstanding the popularity which Koch's theory of the bacterium tuberculosis being the direct and sole cause of tuberculosis, soon acquired after the discovery of this organism, and to a certain extent still holds, it cannot be denied that since that time, so far as may be gathered from the current medical literature, the number of those physicians who doubt the correctness of this theory, has been steadily increasing. Thus some of the most distinguished clinicians, being unable to indorse the whole original theory, have altered it in order to render it harmonious with their own practical experience, and with the old-established fact that tuberculosis is hereditary in its nature, depending upon a special diathesis which is transmitted from the parent to the offspring. Accordingly, Koch's original theory, teaching that the bacterium tuberculosis was capable of producing tuberculosis without regard to predisposition in every individual, has been modified in such a manner as to read now: *Bacterium tuberculosis produces tuberculosis when associated with tubercular diathesis, but is harmless in the absence of the latter; and vice versa, tubercular diathesis leads to tuberculosis if associated with bacterium tuberculosis, but is harmless without this organism.*

Presuming that the reader is familiar with the numerous articles, published in various medical journals, and of which the authors either advocate this theory, or even regard the bacterium tuberculosis as a product of the disease itself, I forbear to enlarge furthermore upon this subject; though I may say that the modification of Koch's original theory rather appears to me as a loss of faith in the capacity of this organism of causing the formation of tubercles. But, notwithstanding the doubts which still exist in regard to this capacity of the tubercle-bacterium, its presence in the tissues of the human lungs, or other organs, must be regarded as pathognomonic of the disease; though cases of tuberculosis are met with in which none of these bacteria are detected in the sputum of the patient, for the reason, that no cheesy centers, containing liberated bacteria tuberculosis, have as yet been formed.

In the foregoing treatise on the relations of the bacterium tuberculosis to the human lungs, or other organs, I have presented the subject to the reader in the light in which it has appeared to me, and, as much as possible, without any prejudice against one or the other theory concerned therein. As far as I know, I have, though unjustly, been regarded as an opponent to the theory of "living germs of disease," because I failed, as in the case of yellow fever, to detect living organisms, to which any signification concerning the cause of the disease might have been attached, in the tissues of the organs which I examined. On the contrary, I have always believed in the possibility of minute living organisms being capable of producing disease by their presence in the blood and tissues, though, in some instances, including tuberculosis, I have doubted the probability of this being the case, for the reason, that the facts, upon which the theory was based, were insufficient to explain all the phenomena manifested by the respective diseases. In those cases, however, where the observed facts covered, or explained all these phenomena, I have never hesitated to acknowledge the facts presented. But, I am unable to enthusiastically receive any new theory, founded upon one or the other observation, on mere faith, though it might be in accord with the already prevailing ideas of the day. Not being naturally inclined to enthusiasm—a passion nearly allied to fanaticism—I prefer in such cases to coolly reflect upon every detail of the subject, and, if possible, to examine it myself, before forming a definite opinion. Every new theory should be received with caution, and not be accepted as true, before it has been thoroughly examined in all its aspects. For nearly thirty years, I have myself been engaged in original studies, but I must confess that, knowing how much every investigator is inclined to interpret some of his observations so as to correspond with one or another favorite idea, I frequently hesitate to draw conclusions from any observation, before I have thoroughly examined and considered the subject in all its aspects. During the progress of science, many theories have been formed, and subsequently abandoned upon the discovery of new and more substantial facts, that explained phenomena which the old theory failed to do. It is, therefore, not impossible, that the same may happen to the now prevailing germ-theory, and that thus the whole problem of the "germs of disease" may be solved in some at present unknown way.

---

### EXPLANATION OF THE ILLUSTRATIONS.

Fig. 1.—Bacteria tuberculosis in tuberculous expectoration; *a*, deeply stained; *b*, feebly stained. Magnified 1090 diameters.

Fig. 2.—Bacteria met with in urine, the description of which will be found in the text.—Magnified 1,090 diameters.

Fig. 3.—Represents a small part of the periphery of a miliary tubercle with several of the neighboring non-tuberculous alveoli; *a*, shriveled nuclei containing more or less bacteria tuberculosis; *b*, epithelial cells of the non-tuberculous alveoli; *c*, inter-alveolar septa with the nuclei of their multiplied connective-tissue cells, derived from the adventitia of their vessels, etc.; *d*, small portion of the periphery of the tubercle; *e*, young tubercle, arisen from the wall of a large alveolus, or perhaps infundibular cavity, and, contrary to the rule generally observed, has undergone the coagulation necrosis.—Magnified 625 diameters.

. Fig. 4.—Represents a portion of a miliary tubercle near its cheesy centre; *a*, bacteria tuberculosis, developing in the nuclei of the tubercle cells; the outlines of the cells are not seen, on account ot the object being mounted in Canada balsam and very highly illuminated; *b*, the same nuclei out of focus; *c*, inter-alveolar septa; *d*, portion of the cheesy centre of the tubercle.—Magnified about 1,090 diameters.

Fig. 5.—Represents a small portion from a large tubercle from the lungs of a boy, sixteen years old, and affected with miliary tuberculosis of the lungs, liver, spleen, kidneys, and lymphatic glands; *a*, bacteria tuberculosis, developed in the nuclei of the tubercle cells; *b*, the same out of focus; *c*, degenerated interstitial connective tissues, leading the central part of the tubercle; *d*, groups of bacteria, commencing to enlarge in their dimensions; *e*, aggregations of bacteria tuberculosis.—Magnified about 1,090 diameters.

Fig. 6.—Bacteria tuberculosis, contained in the nuclei of the muscular and of the connective-tissue cells in the wall of a bronchioles; *a*, nuclei of the connective-tissue cells; *b*, nuclei of the muscular-tissue cells.—Magnified about 1,090 diameters.

Fig. 7.—Bacteria tuberculosis contained in the nuclei of the cells of indurated tuberculous tissue.—Magnified about 1,090 diameters.

Fig. 8.—Represents a small portion of the parenchyma of the liver, bordering directly the periphery of a miliary tubercle; *a*, nuclei of hepatic cells, still presenting a normal appearance; *b*, nuclei with bacteria developing in their interior.—Magnified 625 diameters.

Fig. 9.—Represents a portion of the centre of a miliary tubercle of the liver, having undergone coagulation-necrosis; *a*, fissures, very likely representing the lumen of contracted capillaries; *b*, groups of bacteria tuberculosis with the remains of the protoplasm of the nuclei, in which they were developed; *c*, aggregations of these bacteria.—Magnified 625 diameters.

Fig. 10.—Represents some of the anatomical elements of a miliary tubercle of the liver; *a*, group of normal hepatic cells from near the border of the tubercle; *b*, a pair of hepatic cells from the periphery of the tubercle, the nuclei of which have commenced to shrivel; *c*, a number of nuclei from the cheesy centre of the tubercle, the margins of which are shriveled in a higher degree and broken up into fragments; *d*, ultimate minute fragments of nuclei, some of which have assumed the form of granular filaments.—Magnified about 1,090 diameters.

Fig. 11.—Represents a small portion of a leprous liver; *a*, normal nuclei of hepatic cells; *b*, degenerating nuclei with shriveled margins; *c*, bacteria lepræ, developing in the nuclei of hepatic cells; *d*, bacteria lepræ, liberated by the degeneration of the protoplasm of the nucleus, and disseminating throughout the likewise degenerated protoplasm of the body of the cell.—Magnified 625 diameters.

Fig. 12.—Bacteria lepræ, as met with in the blood of some lepers.—Magnified about 1,090 diameters.

Fig. 13.—Represents a minute portion of the pars reticularis of the corium of the skin from the margin of a leprous tubercle; *a*, nuclei of connective-tissue cells containing bacteria lepræ in their early stage of development; *b*, neoplastic connective-tissue cells in an advanced stage of fatty degeneration, containing fully developed bacteria lepræ between the fat-globules.—Magnified 625 diameters.

Fig. 14.—Represents a minute portion from the middle of the same leprous tubercle, in which the bacteria lepræ are seen in their different stages of development; *a*, bacteria in the nuclei of the unchanged connective-tissue cells ; *b*, bacteria slightly more developed; *c*, the nucleus has disappeared by degeneration and the bacteria lepræ are seen fully developed in the protoplasm of the cell, exhibiting their granular nature ; *d*, fully developed bacteria lepræ between the fat-globules of the degenerated cell.—Magnified 625 diameters.

Fig. 15.—A number of degenerated leprous cells higher magnified, showing to a better advantage the morphological character of the bacteria lepræ.—Magnified about 1,090 diameters.

